# Organellar microcapture to extract nuclear and plastid DNA from recalcitrant wood specimens and trace evidence

**DOI:** 10.1186/s13007-022-00885-z

**Published:** 2022-04-20

**Authors:** Adriana Costa, Giovanny Giraldo, Amy Bishell, Tuo He, Grant Kirker, Alex C. Wiedenhoeft

**Affiliations:** 1 Department of Sustainable Bioproducts, Mississippi State University, Starkville, MS USA; 2 Department of Botany, University of Wisconsin, Madison, USA; 3 Forest Products Laboratory, Madison, WI USA; 4 Department of Wood Anatomy and Utilization Chinese Research Institute of Wood Industry, Chinese Academy of Forestry, Beijing, China; 5 Wood Collections (WOODPEDIA), Chinese Academy of Forestry, Beijing, China; 6 Department of Forestry and Natural Resources, Purdue University, West Lafayette, IN USA; 7 Departamento de Ciências Biológicas (Botânica), Universidade Estadual Paulista–Botucatu, São Paulo, Brasil

**Keywords:** Organellar microcapture, Nucleus isolation, Plastid isolation, Single-cell sequencing, Wood identification

## Abstract

**Background:**

Illegal logging is a global crisis with significant environmental, economic, and social consequences. Efforts to combat it call for forensic methods to determine species identity, provenance, and individual identification of wood specimens throughout the forest products supply chain. DNA-based methodologies are the only tools with the potential to answer all three questions and the only ones that can be calibrated “non-destructively” by using leaves or other plant tissue and take advantage of publicly available DNA sequence databases. Despite the potential that DNA-based methods represent for wood forensics, low DNA yield from wood remains a limiting factor because, when compared to other plant tissues, wood has few living DNA-containing cells at functional maturity, it often has PCR-inhibiting extractives, and industrial processing of wood degrades DNA. To overcome these limitations, we developed a technique—organellar microcapture—to mechanically isolate intact nuclei and plastids from wood for subsequent DNA extraction, amplification, and sequencing.

**Results:**

Here we demonstrate organellar microcapture wherein we remove individual nuclei from parenchyma cells in wood (fresh and aged) and leaves of *Carya ovata* and *Tilia americana,* amyloplasts from *Carya* wood, and chloroplasts from kale (*Brassica* sp.) leaf midribs. ITS (773 bp), ITS1 (350 bp), ITS2 (450 bp), and rbcL (620 bp) were amplified via polymerase chain reaction, sequenced, and heuristic searches against the NCBI database were used to confirm that recovered DNA corresponded to each taxon.

**Conclusion:**

Organellar microcapture, while too labor-intensive for routine extraction of many specimens, successfully recovered intact nuclei from wood samples collected more than sixty-five years ago, plastids from fresh sapwood and leaves, and presents great potential for DNA extraction from recalcitrant plant samples such as tissues rich in secondary metabolites, old specimens (archaeological, herbarium, and xylarium specimens), or trace evidence previously considered too small for analysis.

**Supplementary Information:**

The online version contains supplementary material available at 10.1186/s13007-022-00885-z.

## Background

Illegal logging is a global crisis, impacting both producer and consumer nations; it is estimated that 15 to 30 percent of traded woods are obtained illegally, contributing to deforestation, loss of biodiversity and tax revenue, increased poverty, and social conflicts [1, 2]. A robust approach to combat illegal logging requires integrating different forensic disciplines to answer questions of species identification, provenance, and individual matching [3–5]. Agility in the development of wood forensic technologies is necessary as the list of threatened species is ever-changing as commonly used species become scarce and new species are logged to replace them [6].

The state-of-the-art forensic analysis of wood remains traditional wood anatomy, despite its comparatively coarse taxonomic (typically generic) and geographic (often limited to hemispheric or continental) resolution [7], the restricted nature of such expertise [8], and the time consuming nature of wood anatomical specimen preparation protocols. Of the various available wood forensic technologies, only DNA analysis can be expected to provide identification at the species, population, and individual levels [5]. DNA-based methods also have the advantage that they are comparatively organographically insensitive to the origin of reference materials, and such material (mainly leaves) can be collected without killing an individual plant, which is less true of other methods of wood identification. The extraction of DNA from fresh plant tissues is a routine practice in plant molecular biology and systematics, but such studies almost invariably extract DNA from largely parenchymatous, mechanically soft tissues or organs of the primary plant body (e.g. leaves, developing shoots and roots).

The limiting factor for the overall development and adoption of DNA methods for wood identification is the recalcitrance of wood as a DNA source. In the living tree, most cells comprising wood are the product of programmed cell death, and thus lack nuclei and other organelles at functional maturity [9–11]. Additionally, in many species, the presence of extractives, secondary metabolites associated with heartwood formation, can interfere with the extraction and amplification of DNA. These chemicals are accumulated in both the living cells that synthesize them and adjacent or connected cells into which such compounds are secreted prior to and during heartwood formation [9, 12]. Lastly, as a commercial product, wood is subjected to various industrial processes (e.g., kiln-drying) that can contribute to DNA degradation [10, 12–14]. Thus, typical bulk extraction protocols developed for tissues with a higher proportion of living cells (i.e. leaves, flowers, green stems) are often ineffective in wood [9].

To overcome the low quantity of DNA in wood, many groups have modified their protocols by increasing lysis time [15], attempting to bind polymerase chain reaction (PCR)- limiting chemicals [12, 16], and/or repeating DNA cleaning and concentrating steps [17], in an effort to ensure sufficient quantity and quality of template DNA for PCR amplification. While many authors report success with these methods, they can require comparatively large amounts of sample material and many (often costly) steps to process and purify DNA, thus reducing the types of wood specimens (e.g. trace forensic evidence, cultural property) from which one might expect to extract DNA. It is therefore desirable to develop DNA extraction protocols for wood that are effective for small specimens.

Single-cell sequencing has become an essential tool in medicine, allowing researchers to perform preimplantation genetic diagnosis on human oocytes and embryos produced in vitro [18]. Similar approaches have been developed for cell-specific analysis of plant tissues as well. Karrer et al. [19] developed a method to mechanically puncture fresh, unfixed plant cells with primary walls and aspirate the cell contents for rt-PCR evaluation of mRNA associated with a proton-ATPase, and, importantly, identify a Monte Carlo effect of such small extraction on the likelihood that they recover mRNAs of interest. Kooliyottil et al. [20] used a similar aspiration technique in fresh potato roots to study transcriptional events associated with nematode infection. Subsequent studies employing laser-capture microdissection (LCM) on either fixed and cryotomed [21, 22] or fixed and embedded [23] plant tissue have also achieved tissue- or cell-specific results in plants, typically with organs or tissues from the primary plant body and with primary cell walls, though Blokhina et al. [24] used LCM to study the mRNAs associated with wood formation and cell maturation in cell assemblages in *Picea abies* and *Populus tremula.*

For the purposes of extracting DNA from mature wood for wood identification, an adaptation of the single-cell approach focusing on parenchyma cells could help overcome at least two of the limitations of wood as a substrate. By targeting a single parenchyma cell, focus is placed only on those cells that have DNA available for extraction, thereby reducing the quantity of secondary metabolites. The introduction of PCR-inhibiting secondary compounds can be further reduced by targeting only DNA-containing organelles of interest (e.g. nuclei or plastids) instead of removing the whole cell, thus increasing the amount of DNA per volume or dry mass.

In this study, we report a method, organellar microcapture, to mechanically extract intact nuclei and plastids from parenchyma cells in wood and leaves. We then employ the “spanning” lysis buffer initially developed by Tsuchiya et al. [18] for extracting DNA from single human blastomere cells and demonstrate that adapting single-cell methods can facilitate the extraction and amplification of DNA from nuclei and plastids in wood. We further discuss how this technique could make possible DNA-based forensic analysis of trace botanical evidence or other specimens heretofore considered too small or valuable to process for traditional DNA extraction.

## Results

### Micromanipulation and microcapture

**Fig. 1:**
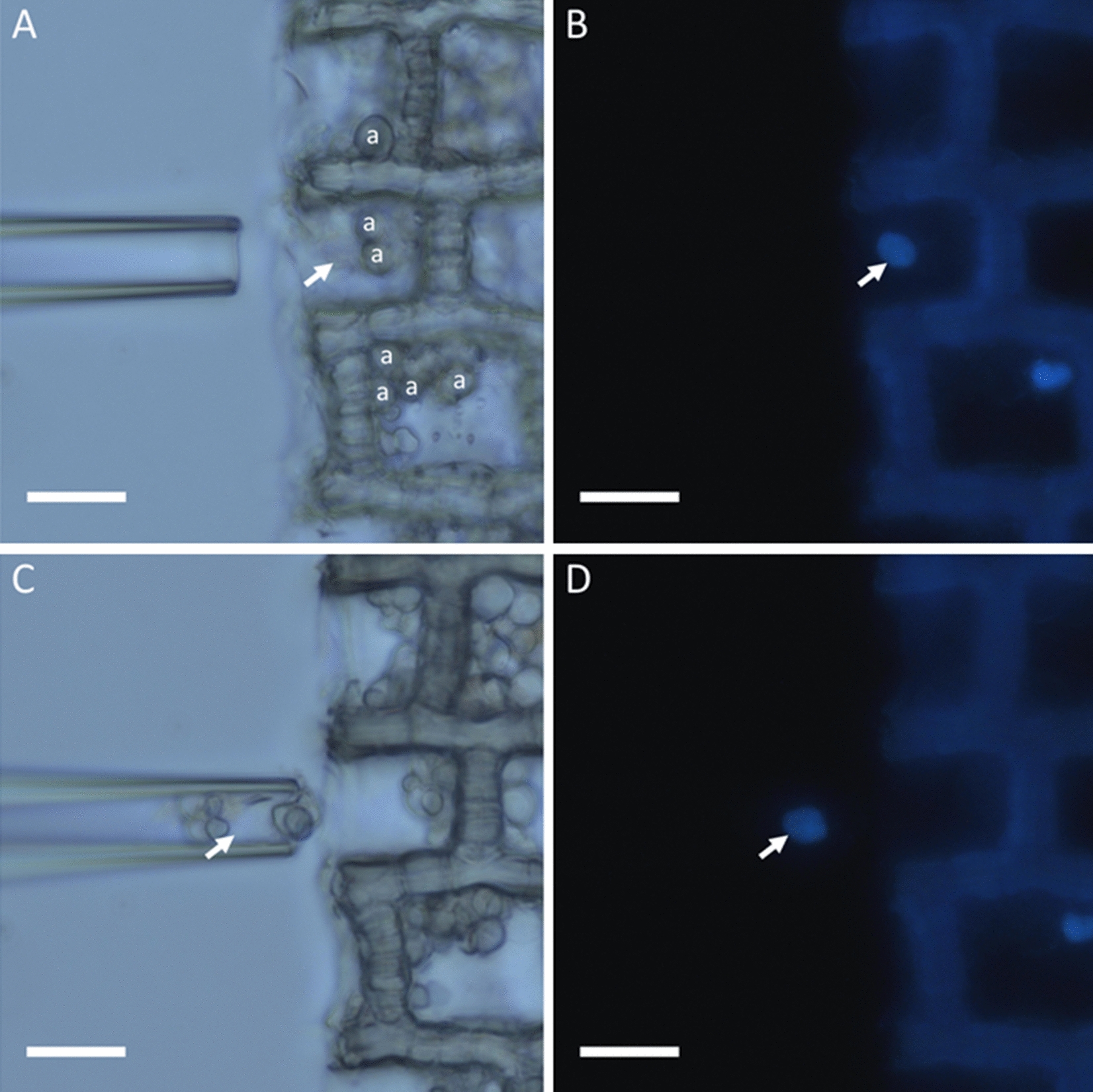
Visualization of organellar microcapture in *Carya ovata* under transmitted light (**A**, **C**) and fluorescence microscopy (**B**, **D**). **A** An empty micropipette prior to insertion into the target parenchyma cell—note that the amyloplasts (**a**) in the target cell and adjacent cells are readily visible, but the nucleus (arrow) is less distinctly resolvable. **B** The same frame as **A**, with the DAPI-stained target nucleus (arrow) easily discernible, and the parenchyma cell walls exhibiting lignin autofluorescence. **C** Amyloplasts and the target nucleus successfully aspirated into the micropipette. **D** The same frame as **C**, confirming that the nucleus is among the captured organelles. Scale bars 20 µ

**Fig. 2:**
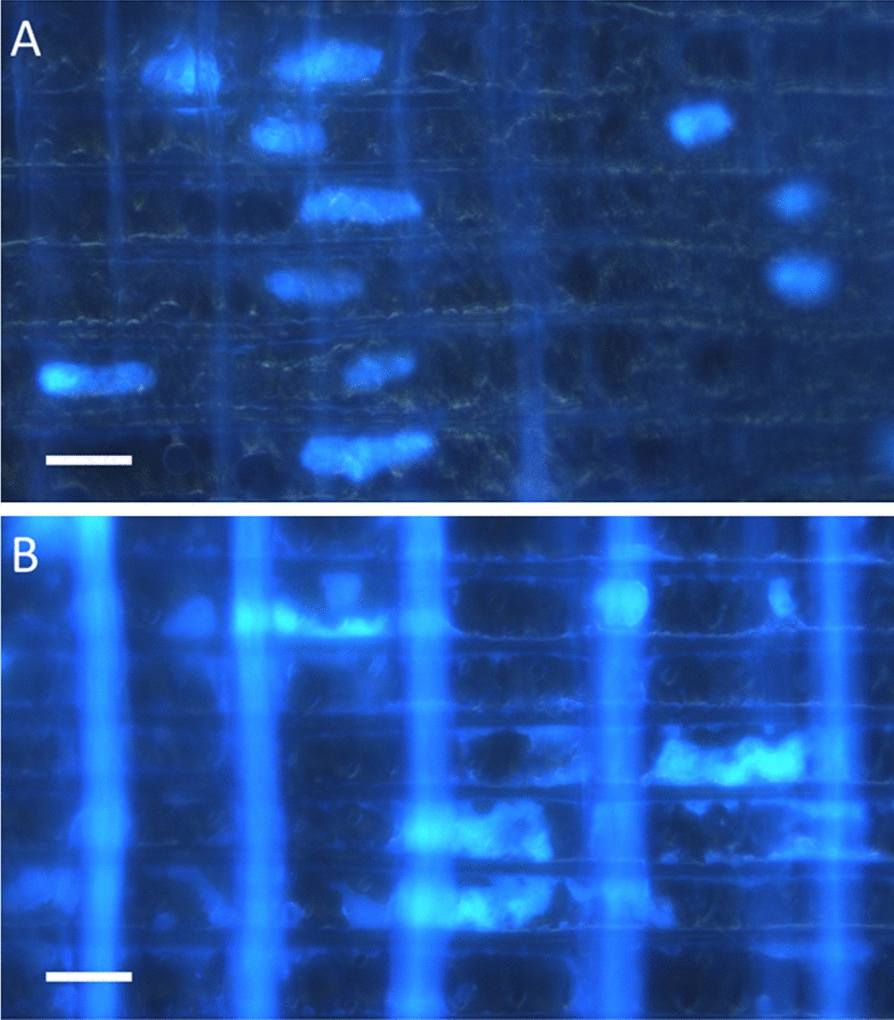
Radial sections (**A**, **B**) in DAPI-stained *Picea* sp. Fluorescent micrographs of intact nuclei (**A**) and dispersed chromatin (**B**) presumably as a result of disruption of the nuclear envelope. Scale bars 30 µm

**Fig. 3:**
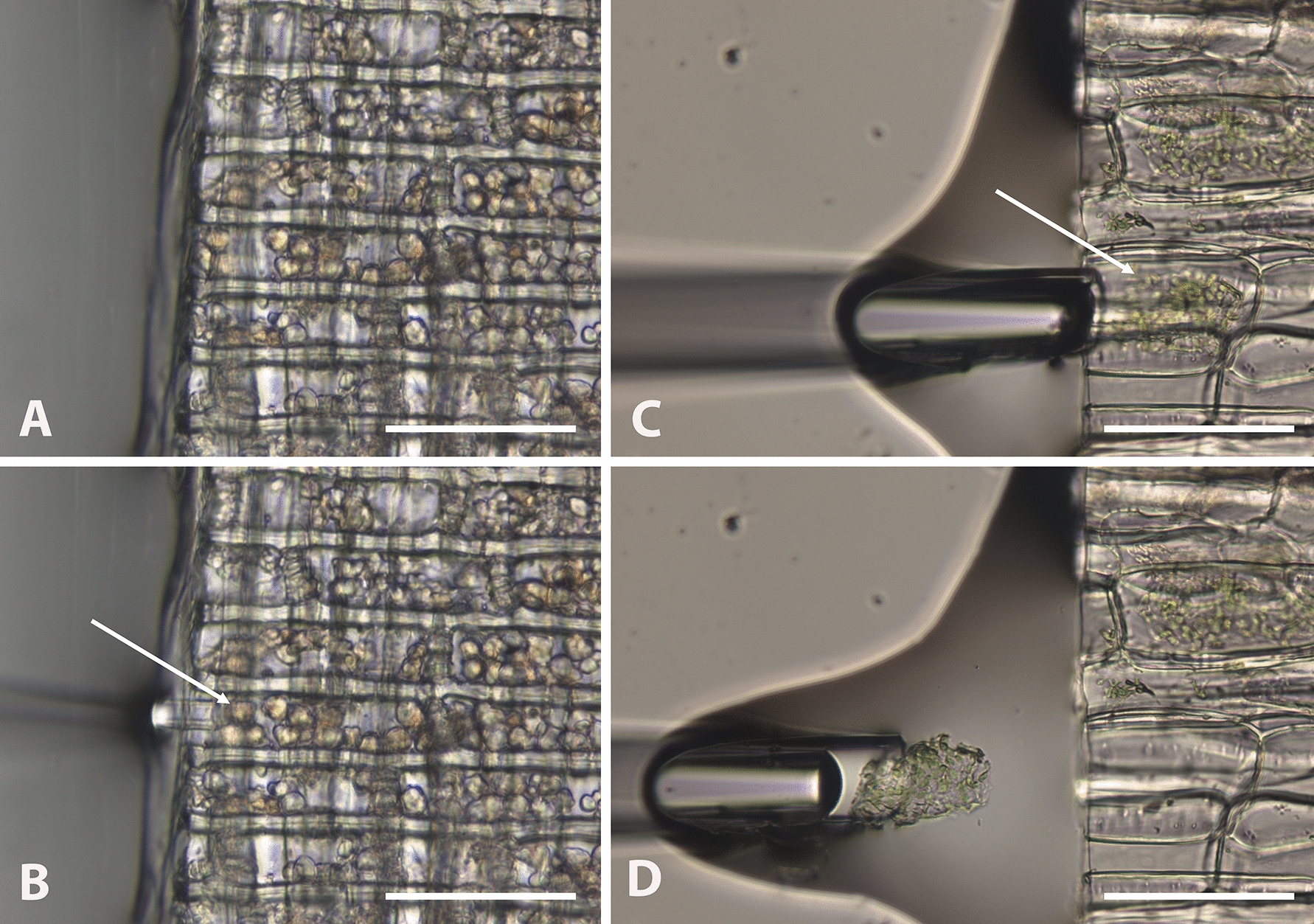
Radial sections (**A**, **B**, *Carya* wood) and longitudinal sections (**C**, **D**, *Brassica* leaf midrib cortical tissue) showing plastids. Amyloplasts are abundant in the ray parenchyma cells (**A**), and in **B** a plastid (arrow) is about to be aspirated into the tip of the pipette. Abundant chloroplasts (**C**) in a slightly plasmolyzed cell (arrow) are aspirated to the pipette tip (**D**), recovering the entire protoplast, ensuring a high copy number of plastids for PCR. Scale bars 50 µm in **A**, **B**, and 100 µm in **C**, **D**

The use of DAPI—4ʹ-6-diamidino-2-phenylindole—allowed easy visualization of intact nuclei during the microcapture process (Fig. 1), and further helped make a subjective inference of nucleus integrity. PCR was often successful with intact nuclei (as seen in Fig. 2A), but when the nuclear envelope was apparently disrupted and the fluorescent chromatin was more dispersed (Fig. 2B), PCR was less frequently successful or failed outright for nuclear microcapture in all taxa studied. Amyloplasts in *Carya* wood and chloroplasts in *Brassica* leaf midribs did not require DAPI for organelle visualization (Fig. 3).

In *Tilia americana*, the nuclei were typically surrounded by a viscous material that in some cases would allow the removal of the nucleus with a thinner pipette tip used as a probe without aspiration. By contrast, in *Carya ovata*, it was typically necessary to dislodge the nucleus mechanically with a thinner simple pipette tip before aspirating it with a microcapture pipette. It was not possible to confirm visually the transfer of nuclei to the PCR tubes.

### DNA polymerase chain reaction (PCR)

PCR products for all regions in both taxa were of the expected sizes, based on the primers used. PCR products from single nuclei from *C. ovata* and *T. americana* are shown in Additional file 1: Figure S1. Internal transcribed spacer—ITS bands were ~ 770 bp, ITS1 ~ 350 bp, and ITS2 ~ 450 bp.

### Sequencing and amplicon verification

Table 1 presents the results of our organellar microcapture protocol in leaves and wood. To ensure the recovered amplicons corresponded to the taxon of origin, heuristic (BLAST—basic local alignment search tool) searches of the sequences were performed against the National Center for Biotechnology Information—NCBI nucleotide database. Organellar microcapture of single nuclei produced successful PCR amplifications and identifiable ITS, ITS1, and ITS2 sequences at the species level for *C. ovata* and *T. americana*. Note that the taxonomy of the GenBank accession does not conform to World Flora Online [25] for *Tilia heterophylla* which is actually a synonym of *T. americana* subsp. *heterophylla*, and where *T. caroliniana* subsp. *heterophylla* is not recognized as a valid scientific name. With respect to this study, recovering generic accuracy is sufficient to demonstrate that the microcaptured organelle was recovered from the taxon of interest. We make no claim that the loci we amplified (ITS, ITS1, ITS2, rbcL) are sufficient for specific identification, but they do suffice for generic confirmation.

**Table 1:** GenBank (genetic sequence database) accession number for the most similar sequence to the amplicon and the corresponding E-value. Note the taxonomy for *Tilia* in GenBank is not congruent with the accepted taxonomy for the genus

Sample	Scientific name in GenBank	Most similar accession in GenBank	E-value
*Carya* fresh leaves ITS	*Carya ovata*	AF174620.1	0
*Carya* fresh leaves ITS1	*Carya ovata*	AF174620.1	4.00E−160
*Carya* fresh leaves ITS2	*Carya ovata*	AF174620.1	0
*Carya* fresh sapwood ITS	*Carya ovata*	AF174620.1	0
*Carya* fresh sapwood ITS1	*Carya ovata*	AF174620.1	1.00E−158
*Carya* fresh sapwood ITS2	*Carya ovata*	AF174620.1	0
*Carya* fresh sapwood rbcL	*Carya illinoiensis*	MN977124.1	9.00E−148
*Carya* MADw 14,525 67y ITS	*Carya ovata*	AF174620.1	0
*Carya* MADw 14,525 67y ITS1	*Carya ovata*	AF174620.1	3.00E−166
*Carya* MADw 14,525 67y ITS2	*Carya ovata*	AF174620.1	0
*Tilia* fresh leaves ITS	*Tilia heterophylla*	AF174639.1	0
*Tilia* fresh leaves ITS1	*Tilia heterophylla*	AF174639.1	0
*Tilia* fresh leaves ITS2	*Tilia heterophylla*	AF174639.1	0
*Tilia* fresh sapwood ITS	*Tilia heterophylla*	AF174639.1	0
*Tilia* fresh sapwood ITS1	*Tilia heterophylla*	AF174639.1	0
*Tilia* fresh sapwood ITS2	*Tilia heterophylla*	AF174639.1	0
*Tilia* MADw 776 75y ITS	*Tilia heterophylla*	AF174639.1	0
*Tilia* MADw 776 75y ITS1	*Tilia caroliniana* subsp. *heterophylla*	KF694723.1	3.00E−143
*Tilia* MADw 776 75y ITS2	*Tilia heterophylla*	AF174639.1	4.00E−172
*Brassica* rbcL	*Brassica rapa*	XM_033273131.1	0

## Discussion

### Micromanipulation and microcapture

*Carya ovata* and *Tilia americana* are angiosperms with moderate amounts of axial and ray parenchyma. Having demonstrated the efficacy of nuclear microcapture in hardwoods and a softwood (see Additional file 1 for a test of the method with *Picea*) spanning low, medium, and high densities with varying ray widths and overall proportions of parenchyma, we expect that this method will be effective for other woods, especially those with more abundant parenchyma, such as *Ficus* spp., or wider rays such as *Quercus* spp. any time an intact nucleus is present and visualizable. We demonstrated plastid microcapture in *Carya* sapwood and chloroplast microcapture in *Brassica* leaf midribs, thus showing that organellar microcapture can be performed when the target organelle, whether nucleus or plastid, is resolvable with the light microscope.

Based on both taxa explored here, the relative ease of, and thus time required for, organellar microcapture varies according to taxon-specific traits. In *C. ovata*, the nuclei were usually adhered to the cell wall (peripheral position), making further manipulation necessary to retrieve them and consequently requiring more time for microcapture. The nuclei of *T. americana* were typically surrounded by a viscous substance that made initial removal easier and faster (around 15 min to extract one nucleus with comparatively little manipulation), but that later appeared to hinder downstream PCR reactions when multiple nuclei were pooled in a single PCR tube (data not shown), though single-nucleus amplification of *T. americana* was successful. Evert & Murmanis [26] reported the presence of tannins, phenolic compounds which have been previously reported as PCR inhibitors [27, 28], in *T. americana* wood parenchyma cells. By contrast, the entire protoplast was readily aspirated from *Brassica* yielding an entire cell complement of plastids—this microcapture required only a matter of 2–3 min.

Microcapture can be made more challenging by cell contents that mechanically impede the aspiration of the organelle. This matter will doubtless be a taxon-specific problem, as observed in *T. americana,* and calls for further research and methodological enhancements, such as using a micropipette to introduce small volumes of solvents or surfactants that could loosen the target organelle without damaging the organellar envelope and the DNA therein. An additional concern is the relative presenting dimension of the organelle, the inner dimension of the cell in which the organelle is located, and the outer and inner diameters of the pipette. If the organelle is of the same size as the inner dimension of the cell wall, it is not possible to fabricate a pipette with an outer diameter narrow enough to enter the cell, but an inner diameter sufficiently large to fully aspirate the organelle. In such cases, mechanically dislodging the organelle and then aspirating it to the tip of the pipette (somewhat analogous to a whole-cell patch-clamp configuration) can suffice to effect organellar microcapture.

### Organelle integrity

Cell maturation for most cell types in secondary xylem involves the deposition of a secondary cell wall, lignification, and programmed cell death, resulting in the absence of organelles. Wood parenchyma cells retain their organelles and remain viable for several to many years in the living tree [29] and nuclear morphology is one of the features employed to infer cell aging processes. Ray parenchyma vitality has been expressed by different indices over the years, including the Nuclear Slenderness ratio (NSR) [30] and the Nuclear Elongation Index (NEI) [31]; in general, these ratios progressively decrease from the outer sapwood to the inner sapwood. Nakaba [32] observed the distribution of cell death in ray parenchyma cells in *Abies sachalinensis* and inferred that the location of a specific radial cell line controls cell maturation and function in a given ray.

In our work, we observed that the microscopic anatomy of the organelle (especially for nuclei) was an important predictor of successful DNA amplification. When we captured chromatin not surrounded by a nuclear envelope, poor to no PCR amplification resulted in all taxa studied, indicating that, in this subjective evaluation of organellar integrity, the technique can only be expected to be successful when one can visualize and extract intact organelles, even from aged wood (see Additional file 1 for results from a 102 year old specimen of *Carya ovata*). We thus presume that DNA obtained from intact nuclei is undamaged relative to its condition in situ prior to microcapture. That is, mechanically capturing a microscopically intact organelle and transferring it to a PCR tube induces the least possible damage to the DNA that we can achieve at this time and therefore represents one method to ensure the minimum possible isolation-induced degradation of DNA quality.

### Nuclear envelope lysis, DNA extraction, and PCR with microcaptured nuclei

Tsuchiya et al. [18] discussed possible reasons for amplification failure from single human cells beyond flawed oligonucleotide primer design, which included failure to transfer the cell, incomplete membrane lysis, ineffective release of DNA from proteins, and degradation or loss of the target sequence.

The transfer of each single organelle into Tsuchiya’s lysis buffer is a step that we could not verify by direct observation, but when PCR amplification was successful and we recovered a BLAST result consistent with the genus of the taxon in question, we considered that the transfer was accomplished. We replicated our procedure three times for each combination of species, plant material, and DNA locus. We recovered positive amplification results in at least one of the replicates, indicating that the PCR was successful, that the organelle was successfully transferred, and lysis and amplification were effective.

Plant DNA extraction kits were developed to overcome the inherent challenges that fresh plant tissues present, such as tannins, phenolic compounds, and complex polysaccharides that can affect DNA quality and inhibit PCR reactions, and typically have focused on leaves as the plant organs most likely to be extracted. However, the methods used for leaves must be adapted for woody tissues because of the lower quantity of DNA, the presence of secondary metabolites, the mechanically tougher lignified cell walls that can be problematic to disrupt, and the likelihood of DNA degradation associated with industrial processing of wood as a material for human use.

Standard DNA extraction kits for plants call for roughly 100 mg to 400 mg of ground tissue for leaves, but leaves have proportionally many more DNA-containing cells than wood. For instance, a leaf can be composed of more than 60% of parenchyma cells and as little as 1% of vascular tissue [33] whereas the proportion of parenchyma cells in wood, although variable, can be as low as 5% [34]. To compensate for this disadvantage, an a priori solution would be to increase the amount of wood in an extraction to increase the DNA yield. However, increasing the amount of wood will also increase the number of cells lacking DNA that may nonetheless release PCR-inhibiting compounds. Similarly, one might expect that increasing the time for lysis might ensure the extraction of all available DNA, but research [15] has shown that simply increasing the amount of wood and/or the time of lysis does not necessarily improve DNA yield. By contrast, the highly targeted capture of single (nuclei) or one to many (plastids) organelles from wood parenchyma cells shown in this study yields reliable results, presumably by excluding most cellular metabolites (primary and secondary) without requiring additional mechanical or chemical processing.

For DNA bulk extraction from leaves we used the Wizard Genomic DNA Purification Kit (Promega, Madison, WI) that required at least 14 steps to extract DNA from plant cells. Kemp et al. [35] state that the manipulation of DNA in traditional DNA extraction methods using commercial kits will result in DNA loss in all steps from extraction to purification. In Tsuchiya et al.’s “spanning protocol,” the only step is to add the nucleus into the lysis buffer and freeze. The resulting solution can be directly used as template for PCR, reducing manipulation mistakes and DNA loss noted by Kemp et al. [35].

### Potential broader applications of organellar microcapture in wood and other plant collections

In addition to its use for recalcitrant specimens in the context of illegal logging, organellar microcapture has the potential to enable the DNA-based characterization of a wide range of previously inaccessible plant materials, because most kits marketed for DNA extraction of plants call for several hundred cells or more of starting material, making it prohibitive when samples are small, degraded, or irreplaceable. Thus any small specimen, whether trace evidence from a crime that would otherwise be expected to yield low DNA quality or quantity [36], or a tiny sample from cultural property (e.g. the wooden cultural patrimony of the Taíno people) [37, 38] might be a source of usable DNA for identification with minimal impact on the integrity of the parent wooden item.

In a similar vein, wood specimens in scientific wood collections may represent one of the only remaining caches of the genetic heritage of now-extirpated populations [39]. Index Xylariorum 4.1 [40] lists approximately 180 xylaria around the world, comprising a substantial number of wood specimens, making these collections valuable resources for accessing genomic information from potentially extinct or extirpated populations. With our technique, picogram quantities of wood from xylaria or leaves from herbaria have the potential to inform the scientific community about targeted genetic characteristics of now-lost genotypes, populations, and perhaps even species.

## Methods

### Overview of the organellar microcapture protocol

**Fig. 4:**
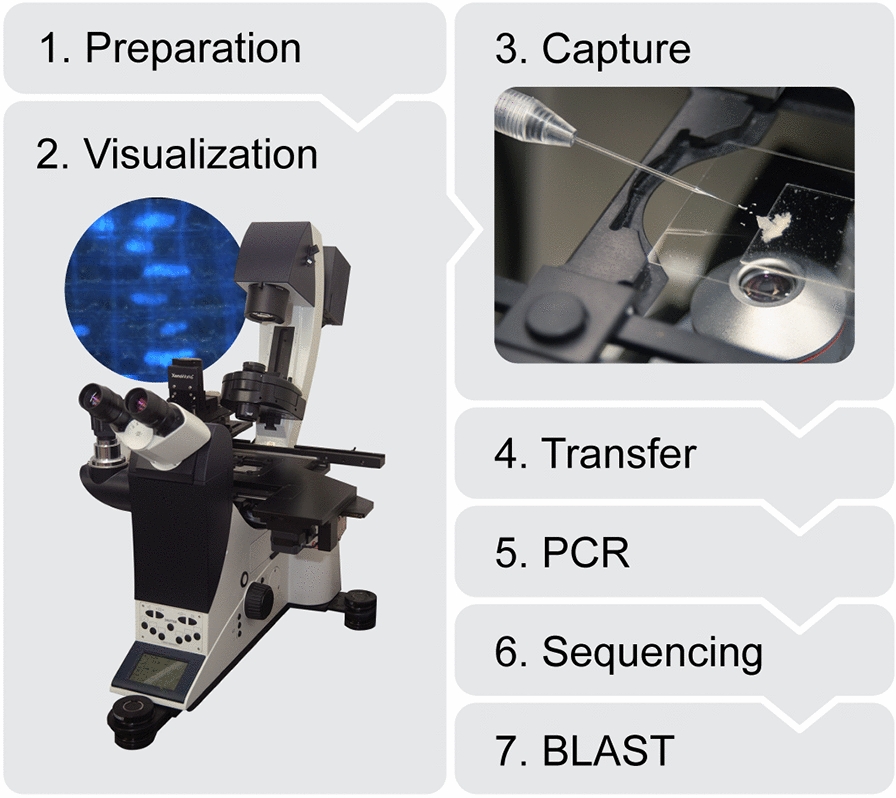
Schematic overview of organellar microcapture protocol

Figure 4 presents a schematic overview of our organellar microcapture protocol comprising seven steps. Details of each step are presented below. Sections (~ 15–25 µm) of wood and leaves were prepared and stained (for nuclei isolation) (step 1) with 0.2% aqueous 4ʹ-6-diamidino-2-phenylindole (DAPI), and visualized with transmitted light and fluorescence microscopy, whereas for plastids DAPI was not required and transmitted light microscopy was sufficient (step 2). At optical microscopy resolutions and magnifications, only nuclei and larger plastids such as chloroplasts and amyloplasts can be resolved individually, the latter of which are sometimes found in sapwood. A micromanipulator was employed to maneuver a pipette to capture the organelles of interest mechanically (adhesion to the pipette) or by aspiration (step 3). The captured organelles were transferred to a PCR tube (step 4) with lysis buffer and then PCR was carried out (step 5). The PCR product was sequenced (step 6), then the resulting sequence was submitted for a BLAST search to confirm that the PCR product corresponded to the taxon from which the organelle was captured.

### Taxon sampling

We selected two angiosperms to develop and test our method in wood: *Carya ovata* (Mill.) K. Koch (Juglandaceae), a medium- to high-density ring-porous hardwood. Fresh samples were obtained from the sapwood of a 255 mm diameter log collected in Richland Center, Wisconsin, on November 04, 2015.

*Tilia americana* L. (Malvaceae), a low-density diffuse-porous hardwood. Fresh samples were obtained from the sapwood of a 300 mm diameter log, collected in Richland Center, Wisconsin, on November 04, 2015.

The logs from the two species were wrapped with clear polyvinylidene chloride and stored at -18ºC at the USDA Forest Products Laboratory-Madison.

To assess the applicability of the method in aged wood, we tested samples of *Carya ovata* and *Tilia americana* retrieved from the Forest Products Laboratory-Madison Wood Collection—MADw. The samples were collected and stored in the xylarium for over 60 years with no specialized preservation method (Table 2).

**Table 2:** Specimens used in this study

Taxon (botanical material)	MADw	Collector name and number	Recorded date of collection	Storage time	Location
				(years)	
*C. ovata* (L)	sn	Adriana Costa/sn	2017	Fresh	Wisconsin, USA
*C. ovata* (FS)	sn	Carl J. Houtman/sn	2015	2	Wisconsin, USA
				(stored under-18 ºC)	
*C. ovata* (AW)	14,525	Robacker, R./F35	1950	67	Pennsylvania, USA
*T. americana* (L)	sn	Adriana Costa/sn	2017	Fresh	Wisconsin, USA
*T. americana* (FS)	sn	Carl J. Houtman/sn	2015	2	Wisconsin, USA
				(stored under -18 ºC)	
*T. americana* (AW)	776	Murray, Frank/sn	1942	75	Michigan, USA
*Brassica* sp. (L)	sn	A.C. Wiedenhoeft/sn	2021	Fresh	Local grocery

Taxon and botanical material, xylarium number (when applicable), collector name and number, recorded year of collection, and location

*L* leaves, *FS* fresh sapwood, *AW* aged wood

Organellar microcapture was also employed on fresh leaves of *C. ovata* and *T. americana,* collected on the University of Wisconsin-Madison campus, and in leaves of kale (*Brassica* sp.) purchased at a local supermarket (Table 2).

Similarly, we tested the microcapture technique on a softwood and on more ancient wood. In **Additional file 1** we show preliminary data on *Picea* sp., a gymnosperm, and a 102 year old sample of *Carya ovata*.

### Sample preparation

Wood: 15 mm sapwood blocks were cut from the specimens. The exposed radial surface was removed with a sliding microtome knife previously cleaned with UltraClean Lab Cleaner (MOBIO Laboratories, Inc, CA, USA), then 15 µm thick radial sections were cut on a sliding microtome, trimmed with a razor blade cleaned as above, and treated with DAPI prior to imaging and microcapture for nuclei, but for amyloplasts from the fresh sapwood of *Carya,* DAPI was not required or used. Leaves: longitudinal hand sections close to the midrib were made and treated with DAPI prior to imaging and microcapture for *Carya* and *Tilia*. In *Brassica* leaves, longitudinal hand sections of the cortical parenchyma of the midrib were made – no DAPI was employed or needed to visualize chloroplasts.

### Micromanipulation and microcapture

We modified the protocol described in Zelinka [41] to convert the apparatus from a probing to an aspiration configuration. Micropipettes were pulled from a void quartz capillary (1 mm O.D. 0.7 mm I.D.) using a programmable laser micro pipette puller [42] using a standard program (Parameters: Heat: 800 Filament: 5 Velocity:60 Delay: 150 Pull: 175). The geometry of the aspiration micropipettes consisted of evenly tapering walls at the base, which transitioned into long parallel walls ending in a 90-degree angle blunt tip. To create the tip a glass-on-glass score-and-break technique was applied to each pipette, as described in Sutter Instrument [42]. The inner diameter was measured, and the pipettes were sorted in pipette boxes by inner diameter (to scale with the diameter of the nuclei for extraction).

As described in Zelinka [41], the micromanipulation system consisted of an inverted microscope (Leica DMI 6000) with a motorized mechanical stage (Leica STP 6000 controlling a Marzhauser EK 127 × 83), a computer controlled three-axis micromanipulator (Sutter Instruments, MPC-200, Novato, CA) and a digital microinjector (XenoWorks BRE Sutter Instruments, MPC-200, Novato, CA). The microscope was fitted with a Grasshopper Express 2.8 MP camera (Point Grey Research, Richmond, BC, Canada) for imaging.

The wood radial sections treated with DAPI were placed on a coverslip (35 × 50 × 1 mm) with DNA-free water to keep them hydrated. For single nucleus extraction, the transfer pressure for microinjection was set to + 80 hPa, and the micropipette was lowered into the liquid. The micropipette was placed next to a ray cell with the lumen exposed (either by sectioning or by trimming with the razor blade), and the nucleus was aspirated into the pipette or held at the tip by lowering the initial pressure as much as needed (down to − 172 hPa) (Fig. 1). The micropipette tip was then dipped into a 0.1 ml PCR tube containing 5 μl of lysis buffer [18], and the nucleus was expelled by increasing the pressure (up to 172 hPa). For leaf longitudinal sections, the same procedure was followed, but instead of targeting ray parenchyma cells, nuclei were aspirated from any open parenchyma cell.

### Nuclear membrane lysis and DNA extraction from microcaptured nuclei

We used the lysis buffer initially developed by Tsuchiya et al. [18] for DNA extraction from single cell human lymphocytes and blastomeres [18]. The lysis buffer was composed of 1 µl H_2_O, 1 µl polyadenylic acid (250 ng/µl), 1 µl EDTA (10 mM), 1 µl dithiothreitol solution (250 mM), and 1 µl N-lauroylsarcosine salt solution (0.5%).

We amplified each region of ITS (ITS, ITS1, ITS2, and rbcL) separately. For each region, we prepared three replicates and added one nucleus to each tube containing 5 µl of cell lysis buffer, totaling nine separate tubes with nuclei captured from wood and nine separate tubes with nuclei captured from leaves, as well as bulk DNA extracted from leaves.

### Bulk leaf DNA extraction

Genomic DNA was isolated from fresh leaves of *C. ovata* and *T. americana* using the Wizard Genomic DNA Purification Kit (Promega, Madison, WI), following the manufacturer’s instructions for plant tissue. We used 20 mg of fresh leaves ground to a powder after freezing in liquid nitrogen.

### DNA polymerase chain reaction (PCR)

For nuclear microcapture in *C. ovata* and *T. americana* the internal transcribed spacer (ITS) and the smaller ITS1, and ITS2 regions were chosen because they are well-sampled across the plant kingdom, with similar logic for the selection of rbcL for plastid microcapture. Plant specific primers for ITS, ITS1 and ITS2 were employed following the designs by Cheng [43] (Table 3). rcbL primers were designed in the NCBI Primer-BLAST tool using the submission tab “primers common for a group of sequences.” The forward and reverse primers were designed using the following target sequences: *Brassica* (accession M88342.1), *Carya* (accession L12637.2), *Hevea* (accession AB267943), and *Daucus* (accession KM360751.1). All ITS PCR reactions were carried out in 25 μl of a reaction mixture containing 5 μl of template DNA (whole volume from the spanning protocol), 5 μl of 5 × Go Taq^®^ buffer (Promega, Madison, USA), 0.4 μl of MgCl2 (25 mM—Thermo Scientific), 1 μl of Bovine Serum Albumin (BSA)(1.25ug/μl), 0.5 μl of dNTP mix (Promega, Madison, USA), 5 μl of each primer (1.0 μM—Custom DNA Oligos Invitrogen), 0.125 μl of GoTaq^®^ DNA Polymerase (Promega, Madison, USA), and 11.975 μl of H_2_O. All PCR reactions were carried out in a DNA thermal cycler (Vapo-Protect, Eppendorf, Germany). For ITS and ITS1, PCR cycles consisted of an initial 2-min denaturation step at 95 ºC, followed by 40 cycles denaturation at 95 ºC (20 s), annealing at 49 ºC (40 s), and elongation at 72 ºC (60 s); then a final 5-min elongation step at 72 ºC. For ITS 2, all the parameters were the same except the annealing temperature was 55 ºC. For rbcL, PCR reactions were carried out in 25 μl of a reaction mixture containing 5 μl of template DNA (whole volume from the spanning protocol), 5 μl of 5 × Go Taq^®^ buffer (Promega, Madison, USA), 0.5 μl of dNTP mix (Promega, Madison, USA), 5 μl of each primer (1.0 μM—Custom DNA Oligos Invitrogen), 0.125 μl of GoTaq^®^ DNA Polymerase (Promega, Madison, USA), and H_2_O to bring the volume to 25 μl. All PCR reactions were carried out in a DNA thermal cycler (Vapo-Protect, Eppendorf, Germany). rbcL PCR cycles consisted of an initial 5-min denaturation step at 94 ºC, followed by 50 cycles denaturation at 94 ºC (20 s), annealing at 55 ºC (40 s), and elongation at 72 ºC (60 s); then a final 5-min elongation step at 72 ºC. A ramp rate of 10% was applied after the annealing step.

**Table 3:** ITS primer sequences developed by Cheng et al*.* (2016) [43] and rbcL primer sequences developed for this study from rbcL sequence data in GenBank

Region	Direction	Sequence
ITS1/5.8S/ITS2	Forward	CCTTATCATTTAGAGGAAGGAG
	Reverse	CCGCTTATTGATATGCTTAAA
ITS1	Forward	CCTTATCATTTAGAGGAAGGAG
	Reverse	GCCGAGATATCCGTTGCCGAG
ITS2	Forward	TGACTCTCGGCAACGGATA
	Reverse	CCGCTTATTGATATGCTTAAA
rbcL	Forward	CAACCATTTATGCGTTGGAGAGA
	Reverse	GGTGCATTTCCCCAAGGGTG

Negative controls (reaction mixture with the addition of an equal volume of DNA-free water as template DNA) were included to ensure the reagents were not contaminated. Three microliters of each PCR product were run in a 1% agarose gel with 0.1 μl/ml SYBR™ Safe DNA Gel Stain (Invitrogen™, CA) alongside a 100-bp DNA ladder (Promega, Madison, USA). Gels were visualized using SmartBlue™ Blue Light Transilluminator (Accuris Instruments, NJ), and bands of the expected size were recorded as positive amplicons.

### Sequencing

The amplification products were purified and sequenced by the Biotechnology Center of the University of Wisconsin-Madison and submitted to run on an ABI 3130xl DNA Analyzer. Alignment and editing of the retrieved sequences were performed in Geneious 11.0.4. (Biomatters Ltd., Auckland, New Zealand). The forward and reverse sequences were aligned into single contigs using the pairwise Geneious Alignment algorithm with default parameters and then manually inspected and trimmed before producing the consensus sequences. To confirm that the obtained sequences were not the result of contamination, we conducted a heuristic search (BLAST) [44] to verify the identity of the consensus sequences.

## Supplementary Information

**Additional file 1**: **Figure S1.** PCR products of microcaptured nuclei from *Carya ovata *and *Tilia americana*. L, Leaf; FS, Fresh Sapwood; AS, Aged Sapwood. **Figure S2.** Fluorescent visualization of organellar microcapture in DAPI-stained *Picea *sp. A. A nucleus partly removed from a ray parenchyma cell. B. The nucleus from A adhered to the tip of a micropipette, and free from the cell. Scale bars 30 μm. **Table S1.** Material description of exploratory samples: taxon and botanical material, xylarium number (when applicable), collector name and number, recorded year of collection, and location. (L) = leaves, (FS) = fresh sapwood, (AW) = aged wood. **Table S2.** PCR primer sequences. 4CL primer sequences developed by Syring et al. (2005) and by our group for species-resolution study in *Picea*. **Figure S3.** Products from the first PCR of microcaptured nuclei from *Carya ovata. *S1, two year old frozen sapwood; S2, 102 year old stored sapwood; S3, 67 year old stored sapwood; C, negative control. **Figure S4.** Products from second PCR round of microcaptured nuclei from *Carya ovata. *S1, two year old frozen sapwood; S2, 102 year old stored sapwood; S3, 67 year old stored sapwood; C, negative control. **Figure S5.** PCR products of bulk leaf extraction and microcaptured nuclei from *Picea *sp. **Table S3.** BLAST search results against the GenBank database for each taxon after organellar microcapture. The GenBank accession number for the most similar sequence to the amplicon and the corresponding E value are provided.

## Data Availability

All data and material generated or analyzed during this study are included in this published article.

## Abbreviations

BLAST: Basic local alignment search tool
DAPI: 4ʹ-6-diamidino-2-phenylindole
EDTA: Ethylenediaminetetraacetic acid
ITS: Internal transcribed spacer
NCBI: National Center for Biotechnology Information
NEI: Nuclear elongation index
NSR: Nuclear slenderness ratio
PCR: Polymerase chain reaction
USDA: United States Department of Agriculture
