# Supplementary material for: Organellar microcapture to extract nuclear and plastid DNA from recalcitrant wood specimens and trace evidence
Source: Plant Methods. 2022 Apr 20;18:51. doi: 10.1186/s13007-022-00885-z (PMC9019980; doi:10.1186/s13007-022-00885-z)
Supplement: Supplementary file 1 — Additional file 1: Figure S1. PCR products of microcaptured nuclei from Carya ovata and Tilia americana. L, Leaf; FS, Fresh Sapwood; AS, Aged Sapwood. Figure S2. Fluorescent visualization of organellar microcapture in DAPI-stained Picea sp. A. A nucleus partly removed from a ray parenchyma cell. B. The nucleus from A adhered to the tip of a micropipette, and free from the cell. Scale bars 30 μm. Table S1. Material description of exploratory samples: taxon and botanical material, xylarium number (when applicable), collector name and number, recorded year of collection, and location. (L) = leaves, (FS) = fresh sapwood, (AW) = aged wood. Table S2. PCR primer sequences. 4CL primer sequences developed by Syring et al. (2005) and by our group for species-resolution study in Picea. Figure S3. Products from the first PCR of microcaptured nuclei from Carya ovata. S1, two year old frozen sapwood; S2, 102 year old stored sapwood; S3, 67 year old stored sapwood; C, negative control. Figure S4. Products from second PCR round of microcaptured nuclei from Carya ovata. S1, two year old frozen sapwood; S2, 102 year old stored sapwood; S3, 67 year old stored sapwood; C, negative control. Figure S5. PCR products of bulk leaf extraction and microcaptured nuclei from Picea sp. Table S3. BLAST search results against the GenBank database for each taxon after organellar microcapture. The GenBank accession number for the most similar sequence to the amplicon and the corresponding E value are provided. [file 13007_2022_885_MOESM1_ESM.docx]

**Additional file.**


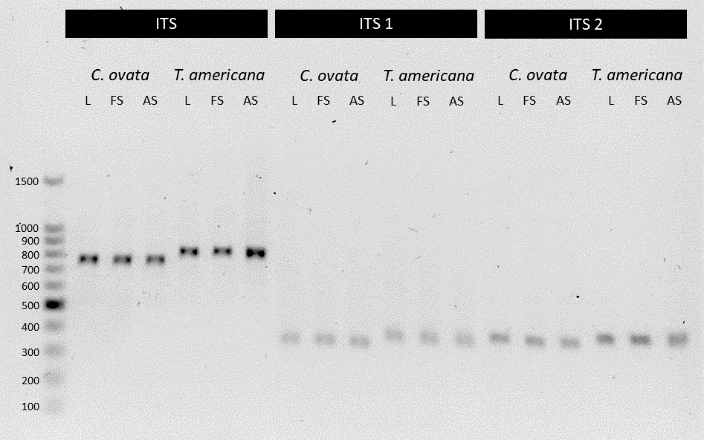


Figure S1. PCR products of microcaptured nuclei from *Carya ovata* and *Tilia americana*. L, Leaf; FS, Fresh Sapwood; AS, Aged Sapwood.

Preliminary data for *Picea* sp. and aged *Carya ovata*.

The microcapture technique was performed in *Picea* sp. (Figure S2) and in a 102 year old sample of *Carya ovata* belonging to the Forest Products Laboratory xylarium (MADw). In Table S1 we present the material description.


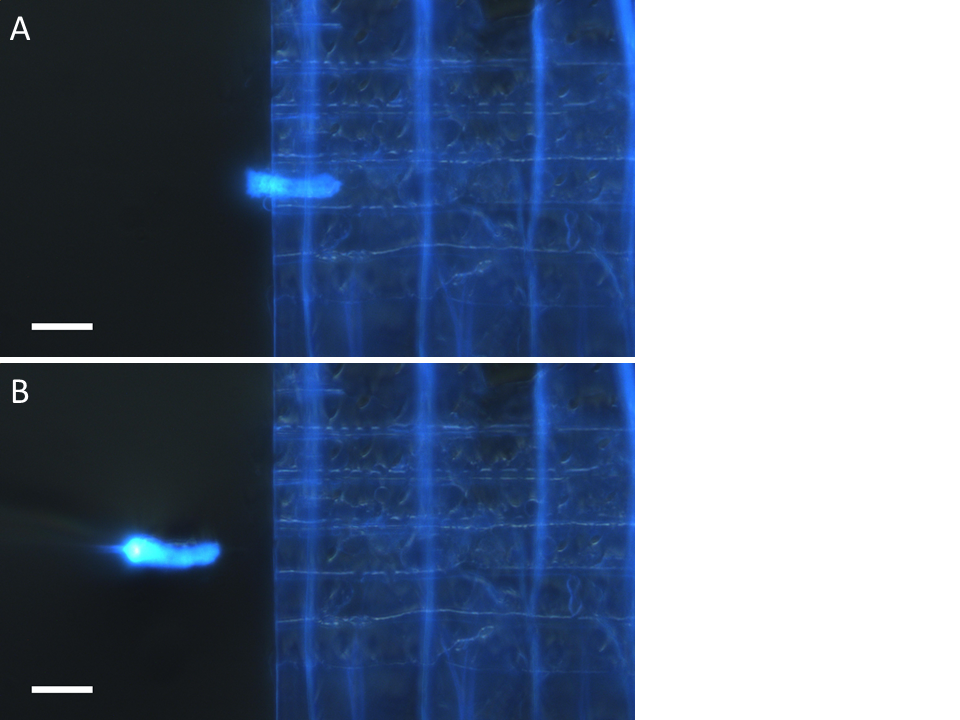


Figure S2. Fluorescent visualization of organellar microcapture in DAPI-stained *Picea* sp. A. A nucleus partly removed from a ray parenchyma cell. B. The nucleus from A adhered to the tip of a micropipette, and free from the cell. Scale bars 30 µm.

| **Taxon (botanical material)** | **MADw** | **Collector name and number** | **Recorded date of collection** | **Storage time (y)** | **Location** |
| --- | --- | --- | --- | --- | --- |
| *Carya ovata* (AW) | 5945 | Koehler, A. / sn | 1915 | 102 | Tennessee, USA |
| *Picea* sp. (FS) | sn | Carl J. Houtman / sn | 2015 | 5, (stored at -18ºC) | Wisconsin, USA |

Table S1. Material description of exploratory samples: taxon and botanical material, xylarium number (when applicable), collector name and number, recorded year of collection, and location. (L) = leaves, (FS) = fresh sapwood, (AW) = aged wood.

For *Carya ovata* we carried-out a successive two-stage PCR to amplify the internal transcribed spacer ITS, ITS1 and ITS2 using the primers developed by Cheng et al. (2016). For *Picea* sp. we amplified the 4CL region, in a single PCR reaction. For leaf bulk extraction, we used *Picea*-specific primers designed by Syring et al (2005) (Table S2). For wood, we used custom designed 4CL primers (Table S2) developed for a species-resolution study in *Picea*.

| **Region** | **Direction** | **Sequence** |
| --- | --- | --- |
| 4CL  (Syring et al. 2005) | Forward | GCCAATCCTTTTTACAAGC |
|  | Reverse | CTGCTTCTGTCATGCCGTA |
| 4CL  (Our group) | Forward | ATTCGGAGGACGTGATTCTG |
|  | Reverse | AATCCGGCAAATGACAGAAA |

Table S2. PCR primer sequences. 4CL primer sequences developed by Syring et al. (2005) and by our group for species-resolution study in *Picea*.

For *Carya ovata* we recovered a band for ITS2 in the first round of PCR but didn’t retrieve visualizable bands for ITS or ITS1 (Figure S3). In a successive second stage PCR we were able to amplify these two regions for aged *Carya ovata* (Figure S4).


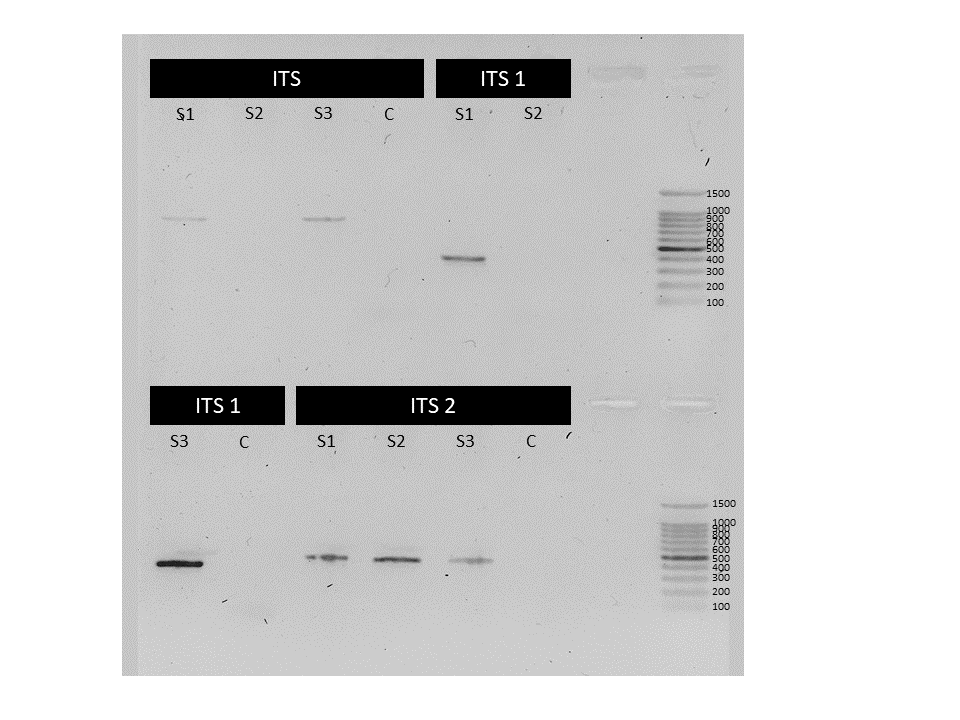


Figure S3. Products from the first PCR of microcaptured nuclei from *Carya ovata.* S1, two year old frozen sapwood; S2, 102 year old stored sapwood; S3, 67 year old stored sapwood; C, negative control.


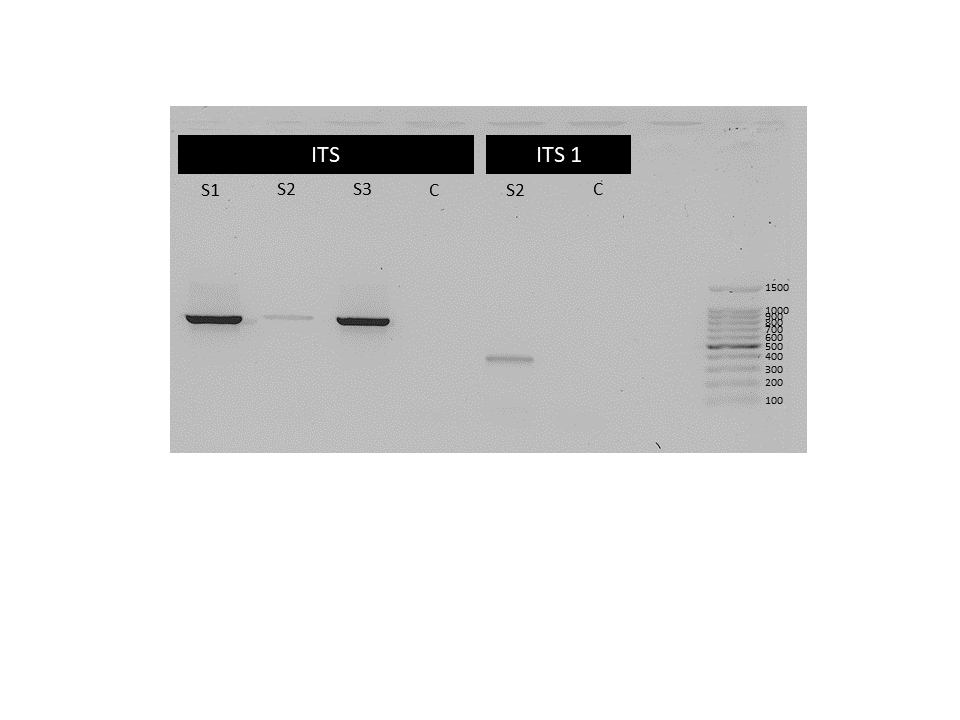


Figure S4. Products from second PCR round of microcaptured nuclei from *Carya ovata.* S1, two year old frozen sapwood; S2, 102 year old stored sapwood; S3, 67 year old stored sapwood; C, negative control.

For *Picea* sp. the single nuclei captured from wood yielded a visible band for 4CL at around 900bp, while the leaf bulk extraction yielded bands of 800bp, which were the expected amplicon sizes for the two different pairs of 4CL primers (Figure S5).


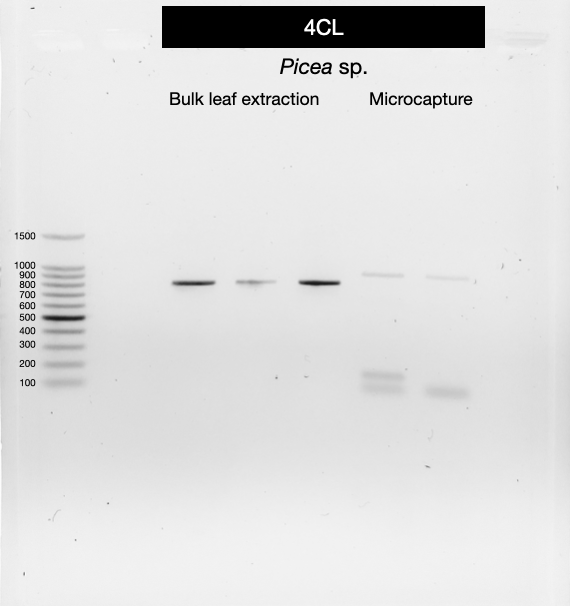


Figure S5. PCR products of bulk leaf extraction and microcaptured nuclei from *Picea* sp.

Table S3 presents the results of our organellar microcapture protocol in leaves and wood of *Picea* sp. To ensure the recovered amplicons corresponded to the taxon of origin, heuristic (BLAST) searches of the sequences were performed against the NCBI nucleotide database. Organellar microcapture of single nuclei produced successful PCR reactions and identifiable ITS, ITS1, and ITS2 sequences, for *C. ovata* (at the species level), and identifiable 4CL sequences at the genus level for *Picea* sp.

| **Sample** | **Name at GenBank** | **Most similar accession in GenBank** | **E-value** |
| --- | --- | --- | --- |
| *Picea* sp. fresh sapwood 4CL | *Picea sitchensis* | JX542521.1 | 0 |
| *Picea* sp. leaves 4CL | *Picea smithiana* | AF144504.1 | 0 |

Table S3. BLAST search results against the GenBank database for each taxon after organellar microcapture. The GenBank accession number for the most similar sequence to the amplicon and the corresponding E value are provided.

**References**

Cheng T, Xu C, Lei L, Li C, Zhang Y, Zhou S. Barcoding the kingdom Plantae: New PCR primers for ITS regions of plants with improved universality and specificity. Molecular Ecology Resources. 2016;16(1):138–49. DOI: 10.1111/1755-0998.12438

Syring J, Willyard A, Cronn R, Liston A. Evolutionary relationships among Pinus (Pinaceae) subsections inferred from Multiple Low-Copy Nuclear loci. American Journal of Botany. 2005;92 (12). 2086-2100. DOI: 10.3732/ajb.92.12.2086.
